# Optic neuritis in German children: clinical findings and association with multiple sclerosis

**DOI:** 10.1007/s00417-020-04669-w

**Published:** 2020-04-10

**Authors:** Felix Tonagel, Helmut Wilhelm, Carina Kelbsch

**Affiliations:** Centre for Ophthalmology, University of Tuebingen, Elfriede-Aulhorn-Str. 7, 72076 Tuebingen, Germany

**Keywords:** Pediatric optic neuritis, Cohort study, Multiple sclerosis, MS

## Abstract

**Purpose:**

Analysis of a cohort of pediatric optic neuritis patients concerning the epidemiology, disease progression, and association with multiple sclerosis (MS).

**Methods:**

Retrospective, observational cohort study. From 2004 to 2018, all electronic medical files of patients younger than 18 years referred to a tertiary care clinic in Germany with the diagnosis optic neuritis have been analyzed.

**Results:**

Sixty-nine patients were referred in the study period, 16 did not suffer under optic neuritis and were excluded. The median visual acuity of the remaining 53 patients was 0.07 at the baseline examination and 1.0 at the latest follow-up examination (decimal notation, median 2.1 years after baseline). Forty-two percent of the patients developed MS during the study period. Female sex (*p* = 0.028) as well as higher age (*p* = 0.0082) proved to be statistically significant risk factors for MS development.

**Conclusion:**

The prognosis for restoring vision in pediatric optic neuritis was favorable. During the observation period, the risk of developing MS was overall 42% and 8% for patients younger than 11 years. The percentage of MS as underlying cause of optic neuritis does not differ remarkably between children older 10 years and adults.

## Introduction

Optic neuritis is an acquired inflammation of the optic nerve and often the first symptom of multiple sclerosis (MS) [1]. The typical patient is a healthy young adult without any remarkable medical history. The gender ratio (women/men) is about 3:1 [2]. In children, optic neuritis is very rare. There are studies of pediatric optic neuritis comprising cohorts of 26 to 59 patients [3–10]. The results of those studies differ considerably: the risk of MS varies from 7.7% in Korea [4] (follow-up 16.3 months) to 17% [8] and 39% [6] in the USA (follow-up 50.4 months and 12 months, respectively). Few data exist from European patients.

In our electronic database, we retrieved all patients younger than 18 years diagnosed with optic neuritis treated in the University Eye Hospital Tuebingen, Germany, from 2004 to 2018.

## Methods

This study was approved by the local ethics committee of the faculty of medicine of the Eberhardt-Karls-University Tuebingen. The study was planned as a retrospective, observational cohort study and within the neuro-ophthalmology unit of the University Eye Hospital in Tuebingen that is a tertiary care ophthalmic clinic.

All electronic medical files of patients younger 18 years with optic neuritis treated from 2004 to 2018 in the University Eye Hospital Tuebingen were analyzed. Sixty-nine minor patients with suspected optic neuritis have been referred to the University Eye Hospital Tuebingen during the study period. The diagnosis of acute optic neuritis was reviewed by neuro-ophthalmologists (the authors) on clinical criteria, considering the presence of subacute visual loss, pain on eye movement, visual field defects, and relative afferent pupillary defect in unilateral cases. Patients with other causes of visual loss than optic neuritis were excluded from further data analysis. Visual acuity values are represented in decimal notation. In cases of younger patients or bad cooperation, we used 90° semi-automated kinetic perimetry as far as practicable, for all other patients 30° suprathreshold automated static perimetry (Haag-Streit Octopus 900 and 101). Visual field defects were divided in central scotoma, defects respecting the vertical meridian, nerve fiber bundle, and unspecific defects. Patients were excluded if the diagnosis could not be confirmed, if other ocular disorders affected the visual acuity or if indications suggested a previous episode of optic neuritis.

All analyses were performed using JMP® 14.2.0 statistical software (SAS Institute, Cary, NC, USA). Continuous data are summarized with the mean and standard deviation, and categorical data are reported as numbers and percentages. To compare continuous unpaired data between groups, the two-sided two-sample *t* test was used. Categorical data were tested using the chi-squared test. A *P* value of less than 0.05 was considered statistically significant.

## Results

The referral diagnosis optic neuritis could be confirmed in 53 patients. In 16 patients, other causes of visual loss were found: uveitis (*n* = 4) and conjunctivitis (*n* = 3) were the most frequent true diagnoses of those patients; posterior scleritis, tension headache, acute zonal occult outer retinopathy, malingering, Leber’s hereditary optic neuropathy, and blunt eye trauma were less common. Painful eye movement, relative afferent pupillary defect (RAPD), and central visual field defects were observed less frequent compared to patients with confirmed optic neuritis (Table 1). The 16 non-neuritis patients were excluded from further data analysis; thus, 53 patients with confirmed acute optic neuritis remained in the study. Mean follow-up was 25.3 months (± 34.3, range 1–126 months), mean age was 12.6 years (± 4.0, range 3.4–17.9), 62% were female, 53% showed white matter lesions in the magnetic resonance imaging (MRI), and 55% had or developed an underlying diagnosis during the study period (Table 2). The majority of the patients exhibited unilateral presentation (72%), RAPD (72%), central visual field defects (64%), and complained about painful eye movements (77%). Unspecific visual field defects (13%) and those respecting the vertical meridian (4%) or complying with nerve fiber bundle (2%) were less common (Table 3). The frequency of RAPD was higher in unilateral patients (84%). The median visual acuity was 0.07 at baseline examination and 1.0 at the latest follow-up examination.

**Table 1:** Differing symptoms of patients with confirmed and excluded diagnosis of optic neuritis

Symptom	Excluded optic neuritis (*n* = 16)	Confirmed optic neuritis (*n* = 53)
Painful eye movement (%^a^)	6 (38)	41 (77)
Relative afferent pupillary defect (%^a^)	6 (43)	38 (72)
Central visual field defect (%^a^)	3 (23)	34 (64)

^a^Total may not equal 100% due to missing data of individual medical files

**Table 2:** Characteristics of pediatric patients with first-episode optic neuritis (*n* = 53)

Mean age (years) (range)	12.6 (3.4–17.9)
Female (%)	33 (62)
Average duration of symptoms prior to presentation (days)	7 (0–30)
MRI of the brain (%)	
White matter lesions present	28 (53)
White matter lesions absent	17 (32)
No data available	8 (15)
Treatment	
Intravenous corticosteroids (%)	32 (60)
Intravenous corticosteroids with oral taper (%)	10 (19)
Steroids combined with intravenous immunoglobulin, plasma exchange or both (%)	3 (6)
No data available	8 (15)
Underlying disease (%)	
Parainfectious	9 (17)
Multiple sclerosis	22 (42)
Acute disseminated encephalomyelitis (ADEM)	2 (4)
Others (neuroretinitis, neurosar-coidosis, sinusitis, anti-aquaporin 4 antibody positive optic neuritis)	5 (9)
No underlying disease identified	9 (17)
Incomplete follow-up data	6 (11)

**Table 3:** Clinical findings of pediatric patients with first-episode optic neuritis (*n* = 53)

Painful eye movement	
Present (%)	41 (77)
Absent (%)	7 (13)
No data available	5 (9)
Unilateral presentation (%)	38 (72)
Swelling of optic nerve head	
Present (%)	27 (51)
Absent (%)	24 (45)
No data available	2 (4)
Relative afferent pupillary defect (all patients)	
Present (%)	38 (72)
Absent (%)	12 (23)
No data available	3 (5)
Relative afferent pupillary defect (unilateral optic neuritis)	
Present (%)	32 (84)
Absent (%)	4 (11)
No data available	2 (5)
Visual field defect	
Central scotoma (%)	34 (64)
Unspecific (%)	7 (13)
Respecting vertical meridian (%)	2 (4)
Nerve fiber bundle (%)	1 (2)
No data available	9 (17)
Oligoclonal bands in cerebrospinal fluid	
Present (%)	15 (28)
Absent (%)	15 (28)
No data available	23 (43)

**Fig. 1:**
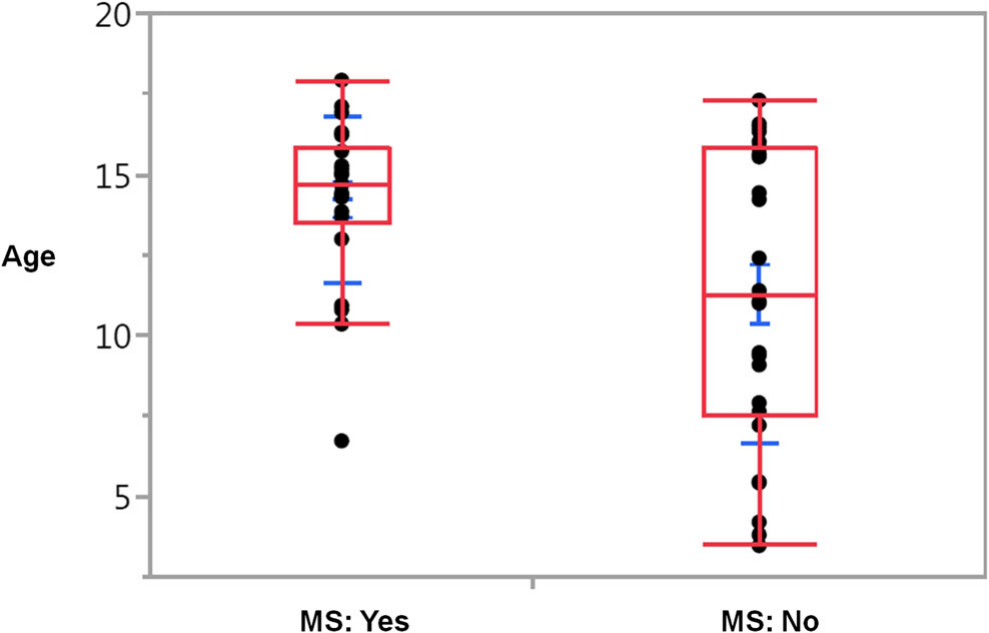
Correlation between age and risk of multiple sclerosis (MS) development (*p* = 0.0082, clinical results for the assessment of a possible MS were available in 48 patients)

**Fig. 2:**
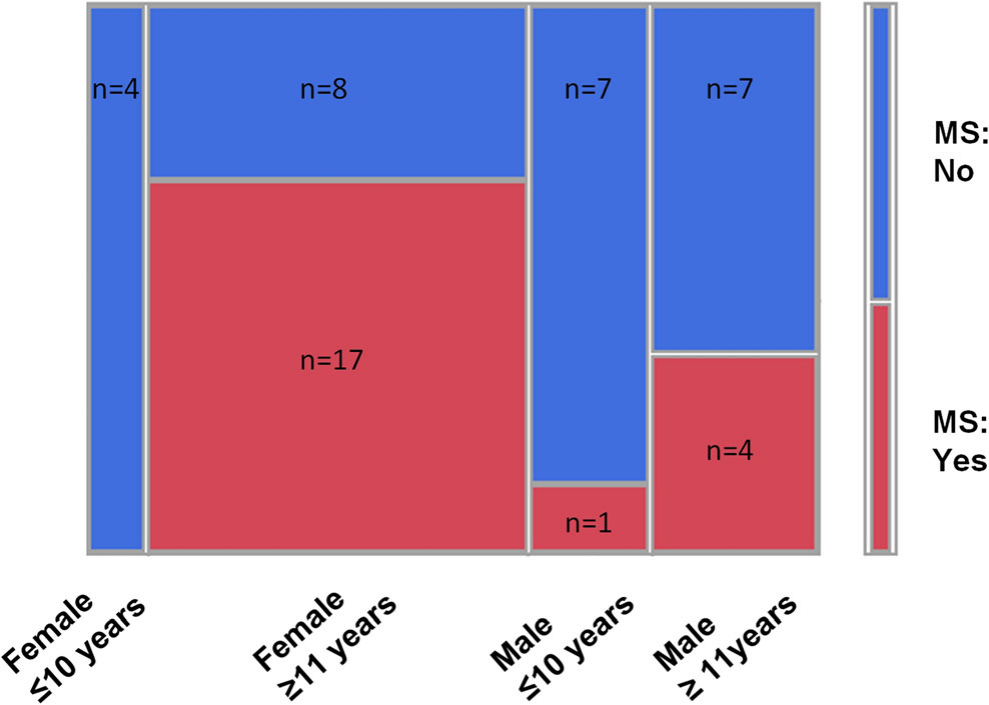
Correlation between age-group/sex and risk of multiple sclerosis (MS) development (*p* = 0.0064, clinical results for the assessment of a possible MS were available in 48 patients)

Sufficient clinical data for the assessment of a possible MS were available in 48 patients. The diagnosis of MS was made by neuropediatricians in accordance with the respectively applicable Mc Donald Criteria and MS-guideline of the German Society of Neurology in 42% of the patients during the study period. Female sex (*p* = 0.028) as well as higher age (*p* = 0.0082) proved to be statistical significant factors which increased the risk of MS development (Fig. 1). For combined consideration of these risk factors, two age groups were formed: group 1 for patients ≤ 10 years and group 2 for patients > 10 years. Female patients older than 10 years had the highest risk of MS development and amounted to 68% (Fig. 2, *P* = 0.0064). Patients younger than 11 years of both genders stood at 8%. Cerebrospinal fluid (CSF) examination was carried out in 30 patients, 14 had positive oligoclonal bands. MRI scans of 43 patients had been evaluated: white matter lesions have been found in 27 patients. Oligoclonal bands and white matter lesions proved to be highly significant risk factors for the development of MS (*p* < 0.001).

## Discussion

Our results confirm a favorable visual prognosis in pediatric optic neuritis. This observation is in accordance with previous work from other authors: Khadse et al. [3] and Kim et al. [4] reported a final visual acuity of ≥ 0.5 for 86% and 77%, respectively. The study of Wan et al. [6] gave account of ≥ 1.0 for 81% of the patients. Nevertheless, a 42% risk for developing MS in our collective lies in the upper range of all reports. Thirty-nine percent reported by Wan et al. [6] and 40.6% by Heussinger et al. [5] are in the same range. By contrast, the conversion rate to MS in Korean children was only 7.7%, but this could be due to the younger study population (median age 10.3 years) and the low incidence of MS in Korea of 0.1 per 100.000 [11] compared to 9.6 per 100,000 in the UK [12]. Obviously, in European and North American patients, MS can be expected in approximately 40%. The highest risk of developing MS affects female patients older than 10 years and stands at 68%. Below that age, the risk was reduced to 8%. The Optic Neuritis Treatment Trial follow-up [1] showed an even higher overall conversion rate to MS of 50% but had a 15-year follow-up and included adults only. Oligoclonal bands and white matter lesions proved to be highly significant risk factors for the development of MS (*p* < 0.001).

Painful eye movement, central visual field defects, and relative afferent pupillary defect (RAPD) are typical symptoms of optic neuritis—their absence may indicate a differential diagnosis. Wilejto et al. [10] reported on the evidence of RAPD in 53% but included bilateral cases for which an RAPD is naturally less likely. In unilateral cases, Wan et al. [6] diagnosed RAPD in 100%. Previous studies did not provide in-depth insight into visual field defects. Kim et al. [4] detected visual field defects in 95%, but did not further classify them. In this respect, the results of our study give insight about the characteristic of visual field defects, which has hitherto not been shown to the best of our knowledge. Further studies seem desirable.

Main limitation of this study is the short follow-up time (median 2.1 years). It can be hypothesized that some more pediatric patients would have developed MS if the follow-up time had been longer. As a conclusion, the percentage of MS as underlying cause of optic neuritis does not differ remarkably between children and adults. Only in children younger than 11 years the MS risk seems to be lower.

## References

[1] Optic Neuritis Study G (2008). Multiple sclerosis risk after optic neuritis: final optic neuritis treatment trial follow-up. Arch Neurol.

[2] Dilokthornsakul P, Valuck RJ, Nair KV, Corboy JR, Allen RR, Campbell JD (2016). Multiple sclerosis prevalence in the United States commercially insured population. Neurology.

[3] Khadse R, Ravindran M, Pawar N, Maharajan P, Rengappa R (2017). Clinical profile and neuroimaging in pediatric optic neuritis in Indian population: a case series. Indian J Ophthalmol.

[4] Kim YM, Kim HY, Cho MJ, Kwak MJ, Park KH, Yeon GM, Lee Y, Nam SO (2015). Optic neuritis in Korean children: low risk of subsequent multiple sclerosis. Pediatr Neurol.

[5] Heussinger N, Kontopantelis E, Gburek-Augustat J, Jenke A, Vollrath G, Korinthenberg R, Hofstetter P, Meyer S, Brecht I, Kornek B, Herkenrath P, Schimmel M, Wenner K, Hausler M, Lutz S, Karenfort M, Blaschek A, Smitka M, Karch S, Piepkorn M, Rostasy K, Lucke T, Weber P, Trollmann R, Klepper J, Haussler M, Hofmann R, Weissert R, Merkenschlager A, Buttmann M, for G-M (2015). Oligoclonal bands predict multiple sclerosis in children with optic neuritis. Ann Neurol.

[6] Wan MJ, Adebona O, Benson LA, Gorman MP, Heidary G (2014) Visual outcomes in pediatric optic neuritis. Am J Ophthalmol. 10.1016/j.ajo.2014.05.036 10.1016/j.ajo.2014.05.036 24907434

[7] Heussinger N, Kontopantelis E, Rompel O, Paulides M, Trollmann R (2013). Predicting multiple sclerosis following isolated optic neuritis in children. Eur J Neurol.

[8] Bonhomme GR, Waldman AT, Balcer LJ, Daniels AB, Tennekoon GI, Forman S, Galetta SL, Liu GT (2009). Pediatric optic neuritis: brain MRI abnormalities and risk of multiple sclerosis. Neurology.

[9] Alper G, Wang L (2009). Demyelinating optic neuritis in children. J Child Neurol.

[10] Wilejto M, Shroff M, Buncic JR, Kennedy J, Goia C, Banwell B (2006). The clinical features, MRI findings, and outcome of optic neuritis in children. Neurology.

[11] Chung SE, Cheong HK, Park JH, Kim HJ (2012). Burden of disease of multiple sclerosis in Korea. Epidemiol Health.

[12] Mackenzie IS, Morant SV, Bloomfield GA, MacDonald TM, O'Riordan J (2014). Incidence and prevalence of multiple sclerosis in the UK 1990-2010: a descriptive study in the General Practice Research Database. J Neurol Neurosurg Psychiatry.

