# Correction to: Optic neuritis in German children: clinical findings and association with multiple sclerosis

**DOI:** 10.1007/s00417-021-05343-5

**Published:** 2021-09-15

**Authors:** Felix Tonagel, Helmut Wilhelm, Carina Kelbsch

**Affiliations:** Centre for Ophthalmology, University of Tuebingen, Elfriede-Aulhorn-Str. 7, 72076 Tuebingen, Germany

**Correction to: Graefe's Archive for Clinical and Experimental Ophthalmology (2020) 258:1523–1526**

10.1007/s00417-020-04669-w

The article **“Optic neuritis in German children: clinical findings and association with multiple sclerosis”**, written by Felix Tonagel, Helmut Wilhelm and Carina Kelbsch, was originally published Online First without open access. After publication in volume 258, issue 7, page 1523–1526, the author decided to opt for Open Choice and to make the article an open access publication. Therefore, the copyright of the article has been changed to © The Author(s) 2020 and the article is forthwith distributed under the terms of the Creative Commons Attribution 4.0 International License (http://creativecommons.org/licenses/by/4.0/), which permits use, duplication, adaptation, distribution and reproduction in any medium or format, as long as you give appropriate credit to the original author(s) and the source, provide a link to the Creative Commons license, and indicate if changes were made. The images or other third party material in this article are included in the article’s Creative Commons licence, unless indicated otherwise in a credit line to the material. If material is not included in the article’s Creative Commons licence and your intended use is not permitted by statutory regulation or exceeds the permitted use, you will need to obtain permission directly from the copyright holder. To view a copy of this licence, visit http://creativecommons.org/licenses/by/4.0. Open access funding enabled and organized by Projekt DEAL.

The original article was corrected.

